# Detection of residual fibroglandular tissue on breast MRI and associated factors in women treated with mastectomy and DIEP flap breast reconstruction

**DOI:** 10.1186/s13244-026-02382-6

**Published:** 2026-08-26

**Authors:** Nieke N. P. M. Smeins, Joep A. F. van Rooij, Esther M. Heuts, Janneke B. Houwers, Patty J. Nelemans, Stefania M. H. Tuinder, Thiemo J. A. van Nijnatten

**Affiliations:** 1 Department of Radiology and Nuclear Medicine, Maastricht University Medical Centre +, Maastricht, The Netherlands; 2 GROW Research Institute for Oncology and Reproduction, Maastricht, The Netherlands; 3 Department of Plastic, Reconstructive, and Hand Surgery, Maastricht University Medical Centre +, Maastricht, The Netherlands; 4 Department of Surgery, Maastricht University Medical Centre +, Maastricht, The Netherlands; 5 SHE School of Health Professions Education, Maastricht University, Maastricht, The Netherlands; 6 Department of Epidemiology, Maastricht University, Maastricht, The Netherlands; 7 Dutch Expert Center for Screening (LRCB), Nijmegen, The Netherlands

**Keywords:** Breast, Magnetic resonance imaging, Mammaplasty, Mastectomy, Perforator flap

## Abstract

**Objectives:**

This study investigates the frequency, extent and location of residual fibroglandular tissue (RFGT) on breast MRI after mastectomy and deep inferior epigastric perforator (DIEP) flap reconstruction. Possible risk factors for RFGT and the association with local breast malignancy after mastectomy (i.e. local recurrence or new primary tumour) are explored.

**Materials and methods:**

This retrospective, single-centre study included patients after mastectomy followed by DIEP flap reconstruction, who underwent breast MRI during follow-up between 2007 and 2022. Breast MRI exams were reassessed for the presence, diameter and location of RFGT. Relative risk was calculated to identify associated factors for RFGT. To investigate the possible association with malignancy after mastectomy, an explorative survival analysis was performed.

**Results:**

Seventy-three patients were included (mean age 45 years, range 21–68), of which 66 were diagnosed with breast cancer. RFGT was detected in twenty (27.4%) patients, with a mean diameter of 13.5 mm (range 6–31), most frequently located in the upper outer quadrant of the breast. Tumour grade 3 seemed to be associated with increased risk of RFGT (RR = 2.7, 95% CI: 1.1–6.6, *p* = 0.050). After a median follow-up time of 110.0 months (range 14–354), malignancy after mastectomy had occurred in thirteen patients; this risk was non-significantly higher in patients with RFGT (HR = 2.3, 95% CI: 0.769–6.827, *p* = 0.137).

**Conclusion:**

RFGT is frequently detected on breast MRI after mastectomy and DIEP flap reconstruction. A baseline postoperative breast MRI exam could possibly be relevant to detect RFGT. The potential association with higher risk of malignancies after mastectomy warrants further investigation.

**Key Points:**

- **Question** research on RFGT detection after autologous reconstruction remains limited; therefore, this study investigates RFGT extent, location, risk factors, and association with malignancy.
- **Findings** RFGT could be detected on breast MRI in 27.4% of patients after DIEP flap reconstruction and might potentially be associated with an increased post-mastectomy malignancy risk.
- **Critical relevance** RFGT can frequently be detected on breast MRI following mastectomy and DIEP flap reconstruction and might be relevant for oncological safety. Future research is needed to validate this and assess the value of a postoperative baseline breast MRI for RFGT detection.

**Graphical Abstract:**

**Figure:**
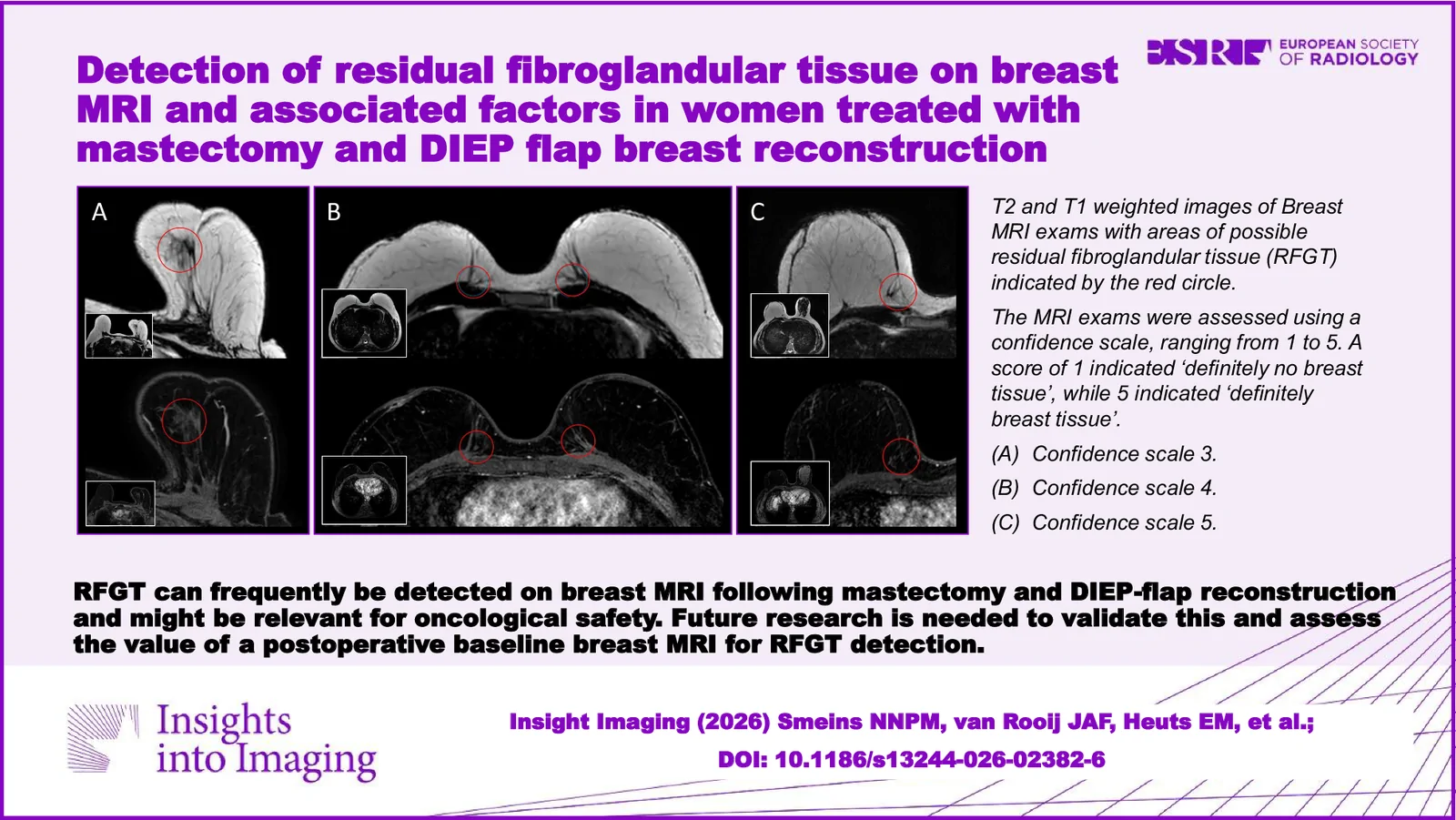

## Introduction

Mastectomy is a surgical procedure designed to remove all fibroglandular tissue from the breast. This procedure can be considered therapeutically or prophylactically [1–3] and is performed in 13.1–35.5% of patients with stages 0 to IIa breast cancer [4–7] and in 18.0–35.7% of high-risk breast cancer patients [8, 9]. Overall mastectomy trends, particularly in terms of surgical rates and treatment choices, vary widely over different countries [4, 6, 10–12].

As mastectomy can harm physical and psychosocial well-being, women increasingly opt for breast reconstruction [13, 14]. Especially, higher rates are seen among younger patients and among those undergoing prophylactic mastectomy [15–17]. Breast reconstruction has been proven to enhance health-related quality of life and psychologic outcomes [18–21]. Autologous reconstruction methods are increasingly being chosen, because of the natural and durable results [13, 22, 23]. The deep inferior epigastric perforator (DIEP) flap is currently the most performed autologous technique [13, 14, 24].

Despite mastectomy being performed as radical treatment, studies report local recurrence risks of 0.6–15.8% [25–27]. After prophylactic mastectomy, breast cancer incidence varies from 0% to 1.9% [1, 28–30]. Previous research suggests that a potential explanation for local recurrences or new primary tumours after mastectomy might be the presence of residual fibroglandular tissue (RFGT) [31]. Magnetic resonance imaging (MRI) has proven to be a valuable tool for detecting RFGT [32–34]. However, according to current international guidelines, there is no indication for radiologic follow-up after mastectomy (and breast reconstruction) [35–37].

As RFGT could pose a risk for oncological safety [32, 38], it might be important to assess the presence of RFGT and contributing factors. Nevertheless, current research on RFGT detection is limited and primarily focuses on implant-based reconstructions or includes all types of breast reconstructions collectively, without clear recommendations on imaging follow-up [32–34]. Given the growing popularity of autologous breast reconstruction and the greater challenges associated with both clinical and imaging-based detection of new malignancies in this population compared with implant-based reconstruction with clearly defined margins or no reconstruction, it may be valuable to study this subgroup specifically.

Therefore, this study aimed to investigate the detection of RFGT presence, extent and location on breast MRI exams after mastectomy and DIEP flap breast reconstruction. Additionally, this study explored potential risk factors for the presence of RFGT, and exploratory analyses were conducted on the effect of RFGT on the risk of malignancy after mastectomy (i.e. local disease recurrence or new primary tumour).

## Methods

### Study design

This was a retrospective single-centre study, performed in the Maastricht University Medical Centre + (Maastricht UMC+) in the Netherlands. The study was approved by the institutional review board of Maastricht UMC+ (METC azM/UM), METC 2022-3122. Informed consent was waived due to the retrospective character of this study.

### Study population

Female patients who underwent therapeutic or prophylactic mastectomy followed by immediate (during the mastectomy procedure) or delayed (in a separate procedure) DIEP flap breast reconstruction were included if a post-reconstruction breast MRI exam was performed between 2007 and 2022 in the Maastricht UMC + . Information on patient, surgery, disease and treatment characteristics was derived from electronic patient files.

### Imaging methods and assessment

In this retrospective study, breast MRI exams were performed on a broad range of clinical indications (i.e. symptoms, follow-up after recurrent breast cancer, etc.). There was no predefined breast MRI exam on a routine basis, and oncological follow-up consisted of physical examination only after mastectomy, without routine imaging surveillance.

Three different MRI systems were used (Intera 1.5 T, Ingenia 1.5 T and Achieva 3 T, Philips Healthcare). A standard breast MRI protocol was used. Details regarding breast MRI protocols are described in Appendix 1.

Evaluation of the reconstructed breasts was performed on axial T2-weighted and axial pre- and post-contrast T1-weighted sequences. For all patients, the first available post-reconstruction breast MRI was used for the assessment.

**Fig. 1:**
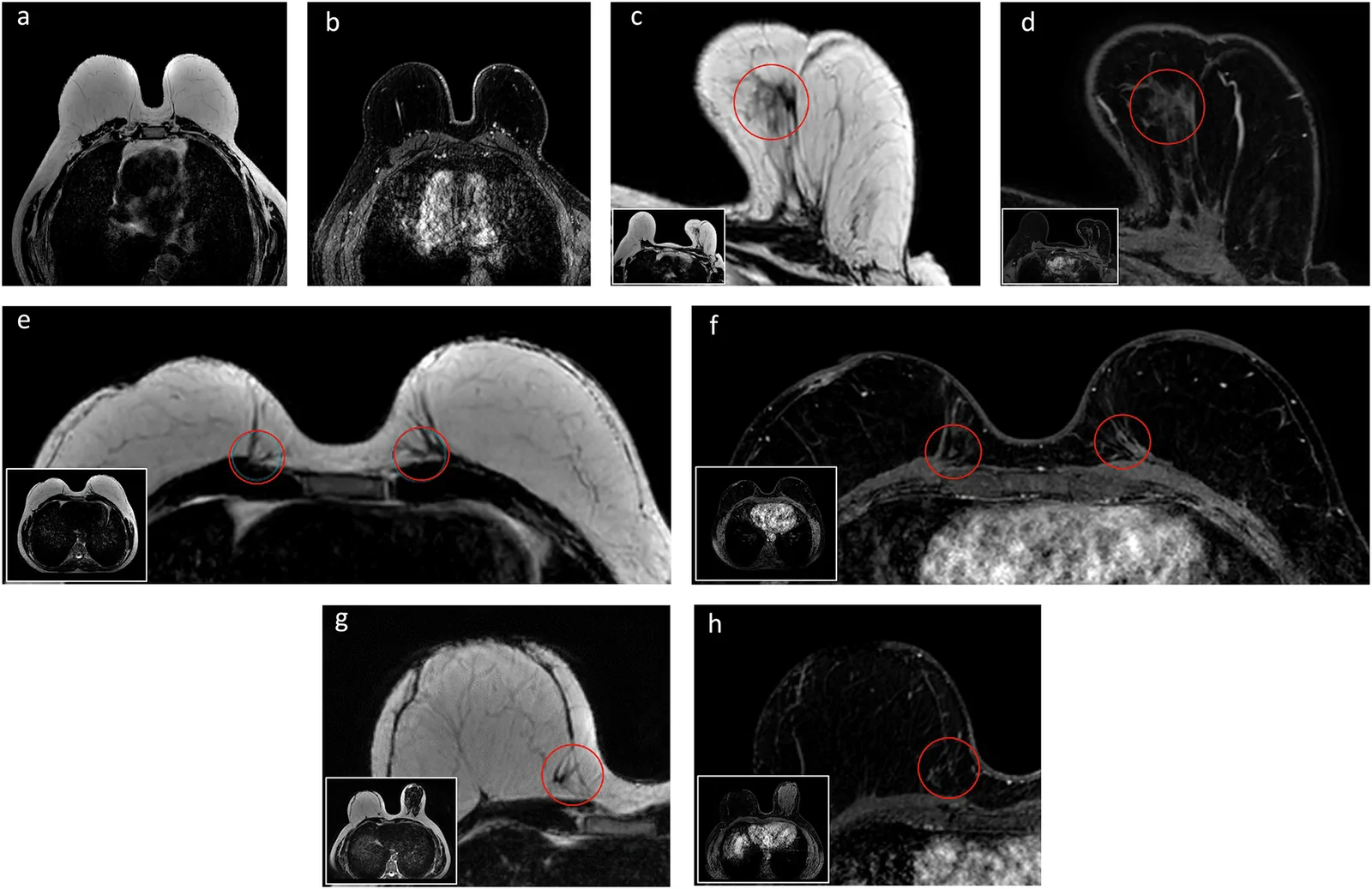
Breast MRI exams assessed for RFGT presence. RFGT is indicated by the red circle if applicable. **a** T2 and **b** T1 weighted breast MRI images of a patient with a bilateral delayed DIEP flap reconstruction, with a confidence score in consensus of 1, because there were no suspect areas for RFGT. **c** T2 and **d** T1 weighted breast MRI images of a patient with a unilateral delayed DIEP flap reconstruction after modified radical mastectomy, with a confidence score in consensus of 3, because of the area located in the upper inner quadrant of the breast. A score of 3 was given because the area could be the result of fibrosis with traction, as well as RFGT. **e** T2 and **f** T1 weighted breast MRI images of a patient with bilateral nipple sparing mastectomy and immediate DIEP flap reconstruction, where RFGT with a confidence score of 4 was located bilaterally in the upper inner quadrants of both breasts. **g** T2 and **h** T1 weighted breast MRI images of a patient with unilateral modified radical mastectomy and immediate DIEP flap reconstruction, where RFGT with a confidence score of 5 was located in the upper inner quadrant of the breast

All breast MRI exams were assessed for the presence of RFGT independently by two dedicated breast radiologists (T.v.N. and J.H.), with 4 and 15 years of clinical experience. As described by Woitek et al [32], RFGT could be recognised by a combination of patchy and linear areas with a relatively hypointense signal, as compared to surrounding fatty tissue. A score form was used to fill out the amount, location and diameter of the RFGT (Appendix 2). The radiologists rated the areas suggestive of RFGT on a confidence scale (1–5), indicating their certainty regarding RFGT involvement. A score of 1 indicated ‘definitely no breast tissue’, while 5 indicated ‘definitely breast tissue’ (Fig. 1). Breast MRI exams rated with a confidence score of 1–3 were considered as no presence of RFGT, while a score of 4–5 was considered as presence of RFGT. The degree of agreement between the radiologists on RFGT presence or absence was calculated in percentages. All analyses were based on consensus assessment of both readers, who discussed discrepancies during a consensus meeting. Histological confirmation of RFGT was not performed.

The location of RFGT was classified based on the four quadrants of the breast. The number of RFGT areas per patient was documented. The size of RFGT was measured in millimetres using the largest diameter. The analysis was performed on a patient level and not on an area level to ensure that observations were independent. In multifocal cases of RFGT, only the RFGT area with the largest diameter was included in the analysis, regardless of whether reconstruction was unilateral or bilateral.

### Statistical analysis

SPSS software (version 28, IBM Corp.) was used for the statistical analyses. Descriptive statistics were used to describe baseline characteristics. Details about disease and treatment characteristics were provided only for patients in whom these factors were relevant, excluding those who underwent prophylactic mastectomy with benign histopathological results and no history of breast cancer.

To explore the association between possible risk factors and RFGT presence, the subgroups of patients with and without RFGT were compared with respect to various patient, surgery, disease and treatment characteristics. Differences between the two groups were tested for significance using the Chi-square or Fisher's exact test (for categorical variables), or the Student’s t-test for independent samples or the Mann–Whitney test (for continuous variables). For each potential risk factor, the relative risk (RR) for RFGT with corresponding 95% confidence interval (95% CI) was calculated. To reduce the risk of excluding potentially predictive variables in this explorative study, a *p*-value < 0.10 was considered to indicate significance. This helps to recognise variables that may be important, but may have been missed using the traditional level of 0.05, especially in small data sets [39].

Exploratory analyses were performed to evaluate whether the presence of RFGT was associated with a higher risk of malignancy after mastectomy. Kaplan–Meier analysis was used [40]. The log-rank test was used to compare survival curves between patients with and without RFGT [41]. Both the 2-year and 5-year cumulative probability of malignancy after mastectomy-free survival were calculated. The proportional hazards assumption was tested. A hazard ratio (HR) for the risk of malignancy after mastectomy with corresponding 95%CI was calculated. Univariate Cox regression analysis was performed [42]. A subgroup analysis was performed for patients with therapeutic mastectomy. The observation period for each patient started at the time of mastectomy. In patients with a malignancy after mastectomy, the observation period ended at the date of the diagnosis of the malignancy. For patients without malignancy after mastectomy, follow-up ended at the date of the last follow-up visit or death, and observations were censored accordingly. The location of the malignancies was compared to the location of the RFGT.

## **Results**

### Patient demographics and surgery characteristics

**Fig. 2:**
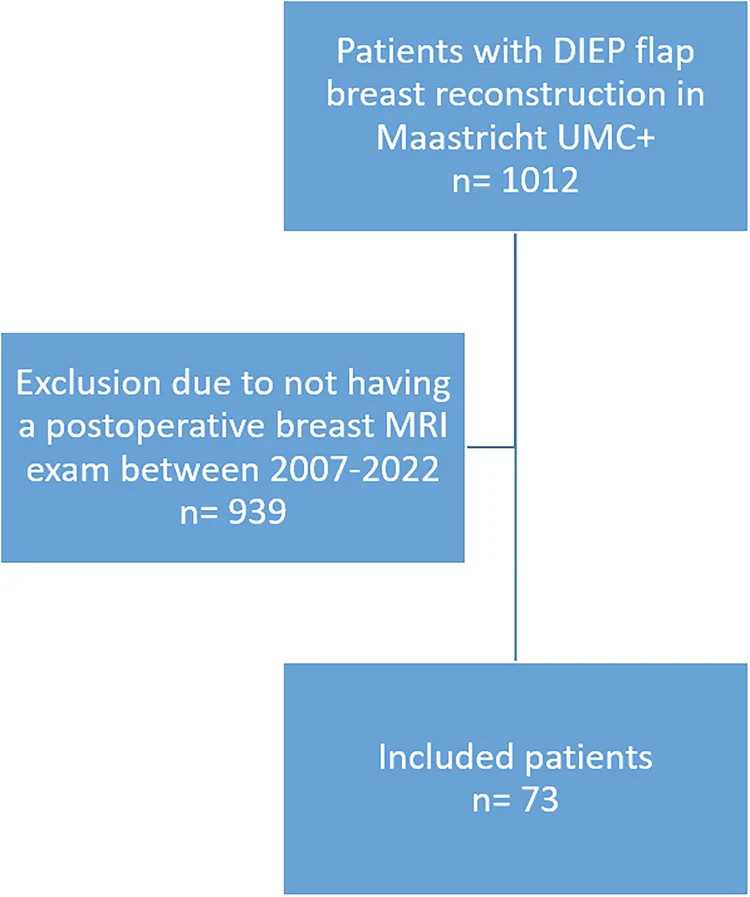
Flowchart of the patient selection process

A total of 73 patients were included (Fig. 2). Mean age at time of diagnosis was 45 years (range 21–68). Mean BMI at time of surgery was 25.3 kg/m^2^ (range 18.4–34.0). In 11 (15.1%) patients, DIEP flap reconstruction was bilateral, resulting in data from 84 breasts.

The indication for mastectomy at the side of the DIEP flap reconstruction was therapeutic in 58 (79.5%) patients and prophylactic in 10 (13.7%) patients. In five (6.8%) patients, mastectomy was performed therapeutically on one side and prophylactically on the other side. The most frequently performed type of mastectomy was the modified radical mastectomy (38.4%). All mastectomies were performed between 1994 and 2019. Immediate reconstruction was performed in 45 (61.6%) patients and delayed reconstruction in 28 (38.4%) patients. In delayed reconstruction, mean time from mastectomy to reconstruction was 44.2 months (range 3-146).

The mean interval between mastectomy and breast MRI exam was 57.0 months (range 7-307). The mean interval between DIEP flap reconstruction and breast MRI exam was 44.1 months (range 4-303).

Gene mutations were present in 18 (24.7%) patients: BRCA-2- (*n* = 8; 44.4%), BRCA-1- (*n* = 6; 33.3%), CHEK-2- (*n* = 2; 11.1%) and PTEN-mutations (Cowden syndrome; *n* = 1; 5.6%).

Further information on patient demographics and surgery characteristics can be found in Table 1.

**Table 1:** Patient and surgery characteristics for patients with and without RFGT

	All patients *n* = 73 (%)	Patients with RFGT *n* = 20 (%)	Patients without RFGT *n* = 53 (%)	*p*-value
Age, mean (range/SD), y	45.36 (21–68)	46.63 (9.659)	44.87 (9.617)	0.489
≤ 40	21 (28.8)	5 (25.0)	16 (30.2)	0.449
> 40	52 (71.2)	15 (75.0)	37 (69.8)	
BMI, mean (range/SD), kg/m2	25.34 (18.4–34.0)	25.00 (4.169)	25.48 (3.829)	0.695
Missing	21 (28.8)			
≤ 25	27 (37.0)	7 (35.0)	20 (37.7)	0.429
> 25	25 (34.2)	8 (40.0)	17 (32.1)	
Mastectomy indication				0.717
Therapeutic	58 (79.5)	16 (80.0)	42 (79.2)	
Prophylactic	10 (13.7)	2 (10.0)	8 (15.1)	
Different for both sides	5 (6.8)	2 (10.0)	3 (5.7)	
Mastectomy type				0.870
Nipple sparing	2 (2.7)	1 (5.0)	1 (1.9)	
Skin sparing	24 (32.9)	7 (35.0)	17 (32.1)	
Simple	10 (13.7)	3 (15.0)	7 (13.2)	
Modified radical	28 (38.4)	7 (35.0)	21 (39.6)	
Radical	1 (1.4)	0 (0.0)	1 (1.9)	
Missing	8 (11.0)			
Laterality DIEP flap				0.479
Unilateral	62 (84.9)	16 (80.0)	46 (86.8)	
Bilateral	11 (15.1)	4 (20.0)	7 (13.2)	
Reconstruction timing				1.000
Immediate	45 (61.6)	12 (60.0)	33 (62.3)	
Delayed	28 (38.4)	8 (40.0)	20 (37.7)	
Familial disease burden^a^				0.562
No	43 (58.9)	11 (55.0)	32 (60.4)	
Yes	21 (28.8)	7 (35.0)	14 (26.4)	
Missing	9 (12.3)			
Gene mutation carriers^b^				0.233
No	55 (75.3)	13 (65.0)	42 (79.2)	
Yes	18 (24.7)	7 (35.0)	11 (20.8)	
Time period of postoperative breast MRI exam				0.093
2007–2010	18 (24.7)	6 (30.0)	12 (22.6)	
2011–2016	21 (28.8)	2 (10.0)	19 (35.8)	
2017–2022	34 (46.6)	12 (60.0)	22 (41.5)	

^a^ First- or second-degree family member with breast cancer

^b^ BRCA-1, BRCA-2, CHEK-2, and PTEN gene mutations

### Disease and treatment characteristics

Breast cancer was diagnosed in 66 (90.4%) patients, three of whom were either diagnosed following an initial prophylactic mastectomy or were diagnosed and treated for breast cancer with lumpectomy prior to prophylactic mastectomy because of a gene mutation. The most common histopathological breast cancer diagnoses were invasive carcinoma of no special type (invasive NST; 60.6%) and invasive lobular carcinoma (9.6%). In 57 (78.1%) patients, lymph node surgery was performed at the time of mastectomy, by either sentinel lymph node dissection (38.4%) or axillary lymph node dissection (39.7%). 25 patients (34.2%) were pathologically classified as N+.

Details regarding disease and treatment characteristics are shown in Table 2.

**Table 2:** Disease and treatment characteristics for patients with and without RFGT

	All patients *n* = 66 (%)	Patients with RFGT *n* = 19 (%)	Patients without RFGT *n* = 47 (%)	*p*-value
Tumour type				0.124
Invasive NST (with or without DCIS)	40^*^ (60.6)	14 (73.7)	26^*^ (55.3)	
Invasive lobular	8^*^ (12.1)	0 (0.0)	8^*^ (17.0)	
DCIS or LCIS	8 (12.1)	2 (10.5)	6 (12.8)	
Other	4 (6.1)	1 (5.3)	3 (6.4)	
Missing	7 (10.6)			
Tumour grade				0.036
1–2	29 (43.9)	5 (26.3)	24 (51.1)	
3	24 (36.4)	11 (57.9)	13 (27.7)	
Missing	13 (19.7)			
Pathological classification				0.574
Primary tumour				
Tx	1 (1.5)	0 (0.0)	1 (2.1)	
T0–2	49 (74.2)	16 (84.2)	33 (70.2)	
T3–4	5 (7.6)	1 (5.3)	4 (8.5)	
Missing	11 (16.7)			
Pathological classification				0.979
Regional lymph nodes				
Nx	3 (4.5)	1 (5.3)	2 (4.3)	
N0	27 (40.9)	8 (42.1)	19 (40.4)	
N+	25 (37.9)	8 (42.1)	17 (36.2)	
Missing	11 (16.7)			
Receptor status				0.756
ER–	18 (27.3)	6 (31.6)	12 (25.5)	
ER+	36 (54.5)	10 (52.6)	26 (55.3)	
				0.765
PR–	24 (36.4)	8 (42.1)	16 (34.0)	
PR+	30 (45.5)	8 (42.1)	22 (46.8)	
				0.351
HER2–	36 (54.5)	9 (47.4)	27 (57.4)	
HER2+	28 (27.3))	7 (36.8)	11 (23.4)	
Missing	12 (16.4)			
Lymph node surgery				0.146
No lymph node surgery	6 (9.1)	4 (21.1)	2 (4.3)	
Sentinel lymph node procedure	28 (42.4)	7 (36.8)	21 (44.7)	
Axillary lymph node dissection	29 (43.9)	8 (42.1)	21 (44.7)	
Missing	3 (4.5)			
Radiotherapy				0.495
No	45 (68.2)	12 (63.2)	33 (70.2)	
Yes	20 (30.3)	7 (36.8)	13 (27.7)	
Missing	1 (1.5)			
Chemotherapy				0.567
None	21 (31.8)	7 (36.8)	14 (29.8)	
Neoadjuvant	12 (18.2)	2 (10.5)	10 (21.3)	
Adjuvant	29 (43.9)	9 (47.4)	20 (42.6)	
Missing	4 (6.1)			
Endocrine therapy				1.000
None	35 (53.0)	10 (52.6)	25 (53.2)	
Adjuvant	28 (42.4)	8 (42.1)	20 (42.3)	
Missing	3 (4.5)			
Immunotherapy				0.286
None	62 (93.9)	17 (89.5)	45 (95.7)	
Neoadjuvant	1 (1.5)	1 (5.3)	0 (0.0)	
Missing	3 (4.5)			

^*^ One mixed case

### Presence, diameter and location of RFGT

For 57 (78.1%) patients, the two readers agreed on the presence or absence of RFGT. Discrepancies on presence or absence of RFGT on breast MRI between the two readers were resolved for 16 (21.9%) patients.

According to the consensus assessment, RFGT was detected on breast MRI in twenty of 73 (27.4%) patients and 21 of 84 (25.0%) breasts. In total, 25 areas of RFGT were found. In one patient with bilateral DIEP flap reconstruction, three areas of RFGT were found (Fig. 1e, f). In three patients, there were two areas of RFGT detected unilaterally.

The mean diameter of the RFGT was 13.5 mm (range 6–31). There was a 4.1 mm difference in the mean diameter of RFGT between patients with immediate and delayed reconstruction (11.8 mm vs 15.88 mm; *p* = 0.194).

RFGT was most frequently located in the upper outer quadrant of the breast (11.0% of patients, 40.0% of RFGT areas). In immediate reconstruction, significantly more RFGT was found in the lower inner and lower outer quadrants of the breast as compared to delayed reconstruction (15.5% vs 0.0%; *p* = 0.022).

**Fig. 3:**
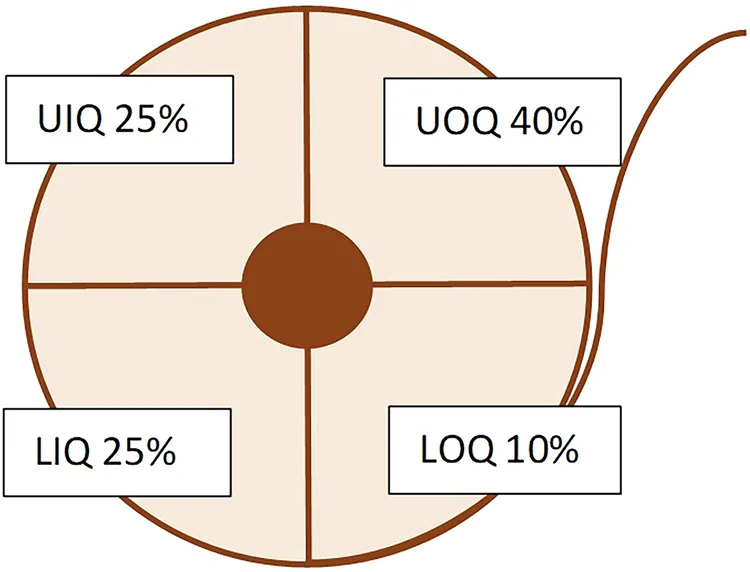
Relative percentages of RFGT location in breast (values of both breasts presented in an example of the left breast; UIQ, upper inner quadrant; UOQ, upper outer quadrant; LIQ, lower inner quadrant; LOQ, lower outer quadrant)

More information regarding RFGT presence, diameter and location, can be found in Table 3. Relative percentages of RFGT location are depicted in Fig. 3.

**Table 3:** RFGT presence, diameter and location in subgroups of patients with immediate and delayed DIEP reconstruction

	Total patients *n* = 73 (%)	Immediate reconstruction *n* = 45 (%)	Delayed reconstruction *n *= 28 (%)	*p*-value
RFGT confidence score ≥ 4	20 (27.4)	12 (26.7)	8 (28.6)	1.000
Diameter of RFGT, mean (range) mm	13.45 (6–31)	10.2 (1–16)	10.9 (7–31)	0.786
Location of RFGT				0.022
Upper inner quadrant	5 (6.8)	2 (4.4)	3 (10.7)	
Upper outer quadrant	8 (11.0)	3 (6.7)	5 (17.9)	
Lower inner quadrant	5 (6.8)	5 (11.1)	0 (0.0)	
Lower outer quadrant	2 (2.7)	2 (4.4)	0 (0.0)	

### Risk factors for RFGT

A higher tumour grade was found to be a statistically significant risk factor for the presence of RFGT (RR 2.7, 95% &&CI: 1.072-6.593, *p* = 0.050). No significant results were found for other potential risk factors. However, mastectomy indication and type, unilateral or bilateral DIEP reconstruction, gene mutation, radiotherapy, lymph node surgery, chemotherapy and pathological tumour classification suggested a tentative association with either increased or decreased RR of RFGT. The RR are presented in Table 4.

### Malignancy after mastectomy

After a median follow-up time of 110.0 months (range 14-354), a malignancy after mastectomy had occurred in thirteen patients. The median follow-up time was 73 months (range 14-165) for patients who developed a malignancy and 131 months (range 18-354) for patients who did not. A malignancy after mastectomy had occurred in both patients with (*n* = 6) and without RFGT (*n* = 7). Out of the thirteen patients with malignancy after mastectomy, three were gene mutation carriers and one had prophylactic mastectomy. In eight of the patients breast MRI was performed after the occurrence of malignancy after mastectomy.

**Table 4:** Relative risks for RFGT presence

	Relative Risk (95% CI)	*p*-value
**Age**		
≤ 40	Ref	
> 40	1.212 (0.505-2.909)	0.899
**BMI**		
≤ 25	Ref	
> 25	1.234 (0.524-2.906)	0.859
**Mastectomy indication**		
Therapeutic	Ref	
Prophylactic	0.725 (0.196-2.681)	0.946
Different for both sides	1.450 (0.458-4.587)	0.889
**Mastectomy type**		
Nipple sparing	Ref	
Skin sparing	0.583 (0.128-2.666)	> 0.999
Simple	0.600 (0.112-3.214)	> 0.999
Modified radical	0.500 (0.109-2.302)	0.938
Radical	-	-
**Laterality DIEP flap**		
Unilateral	Ref	
Bilateral	1.409 (0.580-3.426)	0.697
**Reconstruction timing**		
Immediate	Ref	
Delayed	0.933 (0.437-1.996)	> 0.999
**Familial disease burden**^**a**^		
No	Ref	
Yes	1.303 (0.591-2.874)	0.716
**Gene mutation carriers**^**b**^		
No	Ref	
Yes	1.645 (0.778-3.479)	0.338
**Time period of postoperative breast MRI exam**		
2007-2010	Ref	
2011-2016	0.286 (0.066-1.244)	0.150
2017-2022	1.059 (0.478-2.347)	> 0.999
**Tumour type**		
Invasive NST ( + /- DCIS)	Ref	
Invasive lobular	-	-
DCIS or LCIS	0.714 (0.200-2.549)	0.918
Other	0.714 (0.124-4.106)	> 0.999
**Tumour grade**		
1-2	Ref	
3	2.658 (1.072-6.593)	0.050
**Pathological classification** **Primary tumour**		
T0-2	Ref	
T3-4	0.613 (0.101-3.700)	0.986
**Pathological classification** **Regional lymph nodes**		
N0	Ref	
N+	1.080 (0.478-2.440)	> 0.999
**Receptor status**		
ER-	Ref	
ER+	0.833 (0.360-1.929)	0.905
PR-	Ref	
PR+	0.800 (0.352-1.816)	0.813
HER2-	Ref	
HER2+	1.556 (0.692-3.495)	0.457
**Lymph node surgery**		
No lymph node surgery	Ref	
Sentinel lymph node procedure	0.375 (0.159-0.882)	0.141
Axillary node dissection	0.413 (0.183-0.937)	0.178
**Radiotherapy**		
No	Ref	
Yes	0.625 (0.161-2.422)	0.765
**Chemotherapy**		
None	Ref	
Neoadjuvant	0.500 (0.123-2.032)	0.540
Adjuvant	0.931 (0.413-2.098)	0.863
**Endocrine therapy**		
None	Ref	
Adjuvant	1.000 (0.456-2.194)	> 0.999

^a^ First- or second-degree family member with breast cancer.

^b^ BRCA-1, BRCA-2, CHEK-2, and PTEN gene mutations.

**Fig. 4:**
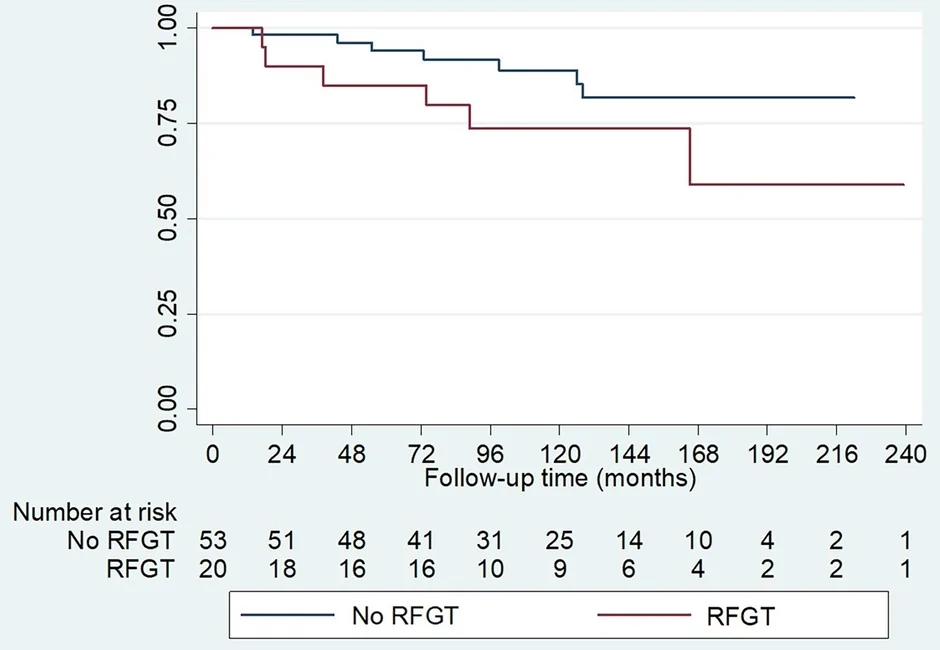
Kaplan–Meier curve for malignancy-free survival after mastectomy according to presence or absence of RFGT

At two years post-mastectomy, the probability of malignancy after mastectomy free survival was 90.0% (95% CI: 0.656–0.974) in patients with RFGT and 98.1% (95% CI: 0.874–0.997) in patients without RFGT. At five years post-mastectomy, probabilities were 85.0% (95% CI: 0.604–0.949) and 94.1% (95% CI: 0.827–0.981), respectively (*p* = 0.126). The HR for malignancy after mastectomy in patients with RFGT vs without RFGT was 2.3 (95% CI: 0.769–6.827, *p* = 0.137). The Kaplan–Meier curve with the numbers of patients at risk during follow-up is shown in Fig. 4. In the subgroup of patients with therapeutic mastectomy, the HR was 1.9 (95% CI: 0.599–5.955, *p* = 0.278).

The mean diameter of RFGT was 12,5 mm in patients with malignancy after mastectomy vs 13.9 mm in patients without malignancy after mastectomy (*p* = 0.690). In the six patients with both RFGT and a malignancy after mastectomy, the tumours were located in the upper inner (2/6), lower inner (2/6) and upper outer (2/6) quadrants of the breast. In four out of six patients in whom both RFGT was found and a malignancy after mastectomy occurred, the location of RFGT matched the location of the tumour.

The histopathology of the subsequent malignancies was comparable to that of the primary tumour in ten patients, while a second primary tumour was observed in three patients.

## Discussion

To the best of our knowledge, this is the first study investigating RFGT in patients who underwent mastectomy and DIEP flap reconstruction. This study aimed to assess the presence, extent and location of RFGT on breast MRI in this specific subgroup. Our results show that in 27.4% of patients RFGT is detected on breast MRI, with a mean diameter of 13.5 mm, most frequently located in the upper outer quadrant of the breast. In addition, we identified a high tumour grade as a possible risk factor for RFGT. In exploratory analyses, a tentative association with a higher risk of malignancy after mastectomy was found in patients with RFGT.

Prior research investigating RFGT on breast MRI in patients after mastectomy and reconstruction found RFGT in 20–100% of cases [38]. While these studies have used varying methodologies, as reflected by the wide range of reported prevalence rates, we applied a structured assessment approach to ensure the best possible consistency, without direct histopathological validation. Although previous research has not specifically addressed the use of breast MRI to evaluate RFGT after autologous reconstruction, our results agree with other studies that proved breast MRI to be a viable modality for identifying RFGT [32–34]. The agreement of 78.1% on RFGT presence between radiologists aligns with these findings, although it might be argued that detection of RFGT can be even further optimised. Specific guidance and training on how to detect RFGT might be valuable. Additionally, artificial intelligence might present a potential opportunity to improve diagnostic accuracy and consistency, although this requires further research.

Distinguishing RFGT from postoperative changes remains challenging. In our study, a confidence scale was used to capture reader uncertainty; nevertheless, the timing of postoperative imaging may significantly influence interpretation. Therefore, in clinical practice, breast MRI findings after surgery should be interpreted with caution, as postoperative changes can persist and may complicate the assessment of RFGT. Moreover, fibroglandular tissue density is known to vary over time, influencing its visibility on imaging, with changes observed during pregnancy, menopause, and after oophorectomy, hormonal and targeted therapy [43]. In our cohort, the mean interval between mastectomy and MRI was 57.0 months. Given that prolonged hormonal suppression may reduce fibroglandular tissue density over time, this could have led to lower RFGT detection rates in some cases [44].

Regarding the location of RFGT, Woitek et al [32] found similar results to ours, with most RFGT located in the upper outer quadrant of the breast. This might be because this area is harder to reach while performing mastectomy using certain incisions. In addition, multiple studies show the retroareolar region to be an important site for RFGT [33, 34, 45, 46]. In our analysis, RFGT found within this region, was classified according to the breast quadrant in which it was located. Another important region identified in the literature is the inframammary fold [47, 48]. Although in this region RFGT is not found as often, the inframammary fold is important for aesthetic outcomes and has been recommended to be preserved during immediate reconstruction to facilitate breast reconstruction [47, 49]. Supporting this recommendation, we found a higher prevalence of RFGT in the lower half of the breast following immediate reconstruction, as compared to delayed reconstruction. Awareness of these zones is important for surgical planning to minimise the risk of RFGT [50].

We found that nipple-sparing mastectomy may be associated with a higher risk of RFGT, although our results were not significant. Previous research provides evidence that supports this potential relationship [32]. In addition to the relatively high baseline density of fibroglandular tissue in the retroareaolar region, preservation of the skin-envelope and nipple-areolar complex in this procedure likely contributes to the RFGT in this region and explains the widely reported observation that RFGT is frequently located behind the nipple [51]. This is important to note, for both surgical planning and imaging follow-up.

The only significant risk factor for RFGT identified in our study was a high tumour grade. This finding is unexpected, as it could be hypothesised that higher tumour grades might prompt a more thorough surgical approach to ensure maximal tissue removal. Conversely, it could also be argued that the presence of a high-grade tumour may present greater surgical complexity, for example due to less favourable tumour characteristics or more challenging breast anatomy, which could increase the likelihood of RFGT. However, since breast MRI exams were not routinely performed, only patients for whom an MRI was clinically indicated were included in this cohort. Grade 3 tumours are known to have a higher risk of recurrence [52], which may have led to more frequent clinical indications for breast MRI exams in these patients. As a result, grade 3 tumours may be overrepresented in this cohort, potentially leading to a selection bias toward this group. Higher tumour grades have not been described as risk factors for RFGT in other literature. Future studies are needed to further explore this association and to better elucidate the underlying biological and surgical factors involved.

Notably, in our study, therapeutic mastectomy was associated with a potentially higher risk of RFGT compared to prophylactic mastectomy, a finding that contrasts with the results of prior studies [32, 34, 46, 53]. In addition to prophylactic mastectomy, other studies identified higher volumes of RFGT in younger patients [51]. Although the literature is limited and outcomes remain inconsistent, these findings may be concerning, as patients with breast cancer at a young age and prophylactic mastectomy (often because of genetic predisposition) are also patients at high risk for (recurring) disease [54]. Furthermore, younger age and prophylactic mastectomy are associated with the choice for breast reconstruction, which on its own has been associated with higher RFGT prevalence [15–17, 34, 51, 55]. Considering the possible association between RFGT and young age, prophylactic surgery and breast reconstruction, as well as the high a priori risk for these patients, accurate diagnosis of RFGT in this subgroup of patients could hold significant clinical importance.

Other risk factors for RFGT identified in the literature include surgeon (experience) [46, 56], age [33, 51], skin flap thickness [56–58], patient height [38], larger preoperative baseline volumes [38, 57] and high breast density [56]. These findings suggest that individual patient characteristics, surgical approaches, and reconstruction techniques all play a role in the extent of RFGT. Understanding these risk factors is critical for tailoring surgical strategies to minimise RFGT and associated risks.

Previous studies highlight the importance of reporting RFGT in post-mastectomy imaging [33, 38, 53]. A recent study by Fritsch et al [59] states that pre- and post-mastectomy breast MRI exams are beneficial to avoid RFGT. Papassotiropoulos et al [46] state that subgroups of high-risk patients might benefit from radiologic follow-up post-mastectomy, which includes detection of RFGT.

A tentative association between the presence of RFGT and higher risk of malignancy after mastectomy was observed in our study, although these analyses were exploratory. Demonstrating a causal relationship would require adjustment for all relevant confounders, which was not feasible due to the low number of events. The survival analysis is further limited by important methodological constraints. RFGT is assessed at the time of the first available MRI, which varies after mastectomy and, in several cases, occurs after the development of malignancy, introducing potential bias. Moreover, the cohort is selected due to clinically indicated imaging, and event rates appear high compared with unselected post-mastectomy populations. Therefore, the association between the presence of RFGT and risk of malignancy after mastectomy should be interpreted with caution. Nevertheless, research by Deutschmann et al [45] supports the plausibility of the relationship between RFGT and development of malignancy after mastectomy. Their study found that higher volumes of RFGT lead to more frequent local recurrences and new primary tumours. Conversely, Kaidar-Person et al [60] found no association between RFGT and risk of locoregional recurrence or any other breast cancer event. However, subcutaneous fat was included in their measurement of RFGT, resulting in relatively high RFGT volumes.

### Limitations

The sample size of our study was relatively small, and not all information on patient, disease and surgery characteristics was available for every patient, as for some patients, initial treatment of breast cancer took place in other hospitals. This limited the power to detect significant associations with risk of malignancy after mastectomy and to control for multiple potential confounders with multivariate analyses.

In addition, several factors may have influenced the assessment of RFGT. First of all, the breast MRI exams were not of equal quality due to the wide timeframe in which they were performed, yet were considered to be of sufficient diagnostic value by the readers. Although differences in field strength and system generation may affect image quality, standardised protocols and the use of dedicated coils allow for a comparable assessment of RFGT across systems [61]. Secondly, it was observed that the anastomosis of the internal mammary artery with the DIEP flap was challenging to assess on breast MRI, making it difficult to distinguish this area from RFGT. Thirdly, specific surgical approaches used in this single-centre study may have affected the prevalence of RFGT. Fourth, RFGT assessment was not verified by pathological examination. Although assessment was performed by experienced breast radiologists, the reliability of RFGT assessment cannot be confirmed. Additionally, many breast MRI exams were performed after local recurrences, or new primary tumours had been diagnosed and treated. Removal of RFGT during treatment may have resulted in underestimation of the presence of RFGT. Underestimation of RFGT presence might have led to weaker associations in this study [62].

Lastly, the 2-year and 5-year probability of malignancy after mastectomy in this study population are quite high, which may be explained by the fact that all breast exams were performed based on clinical indications rather than routine imaging. Nevertheless, the selection of a high-risk population is unlikely to have consequences for the comparison of the relative risk of recurrence between patients with and without RFGT.

### Future perspectives

The regular occurrence of RFGT after mastectomy and DIEP reconstruction highlights the importance of increased awareness of RFGT. Since this often involves relatively young patients with long-term follow-up, considering baseline imaging following autologous reconstruction might be interesting in future clinical practice. A first step might be to advise radiologists to report on RFGT. Given the limited literature on RFGT, both in terms of the number of studies and sample sizes, it is important to conduct further research in larger multicentre studies to confirm findings. Especially to investigate the association between RFGT and the risk of developing a local malignancy after mastectomy, as well as to enable subgroup analyses in high-risk patients. We hope that our study contributes to increased awareness of this important topic and may help stimulate further research in this field.

### Conclusion

RFGT can frequently be detected on breast MRI exams after mastectomy and DIEP flap breast reconstruction. Future studies should focus on the clinical consequences of the visualisation of RFGT on breast MRI. Especially the possible association between RFGT and risk of malignancy after mastectomy, and whether there is a role for breast MRI in the follow-up of post-mastectomy patients with subsequent autologous breast reconstructions in the case of RFGT should be further investigated.

## ELECTRONIC SUPPLEMENTARY MATERIAL

Appendix 1

Appendix 2

## Data Availability

The datasets generated and/or analysed during the current study are not publicly available.

## Abbreviations

DIEP: Deep inferior epigastric perforator flap
RFGT: Residual fibroglandular tissue

## References

[1] Heemskerk-Gerritsen BA, Menke-Pluijmers MB, Jager A et al (2013) Substantial breast cancer risk reduction and potential survival benefit after bilateral mastectomy when compared with surveillance in healthy BRCA1 and BRCA2 mutation carriers: a prospective analysis. Ann Oncol 24:2029–2035 23576707 10.1093/annonc/mdt134

[2] Hartmann LC, Sellers TA, Schaid DJ et al (2001) Efficacy of bilateral prophylactic mastectomy in BRCA1 and BRCA2 gene mutation carriers. J Natl Cancer Inst 93:1633–1637 11698567 10.1093/jnci/93.21.1633

[3] Simons JM, Jacobs JG, Roijers JP et al (2021) Disease-free and overall survival after neoadjuvant chemotherapy in breast cancer: breast-conserving surgery compared to mastectomy in a large single-centre cohort study. Breast Cancer Res Treat 185:441–451 33073303 10.1007/s10549-020-05966-y PMC7867515

[4] Garcia-Etienne CA, Tomatis M, Heil J et al (2012) Mastectomy trends for early-stage breast cancer: a report from the EUSOMA multi-institutional European database. Eur J Cancer 48:1947–1956 22483323 10.1016/j.ejca.2012.03.008

[5] Fancellu A, Sanna V, Cottu P et al (2018) Mastectomy patterns, but not rates, are changing in the treatment of early breast cancer. Experience of a single European institution on 2315 consecutive patients. Breast 39:1–7 29454174 10.1016/j.breast.2018.02.003

[6] Fefferman M, Nicholson K, Kuchta K, Pesce C, Kopkash K, Yao K (2023) Rates of bilateral mastectomy in patients with early-stage breast cancer. JAMA Netw Open 6:e2251348 36652251 10.1001/jamanetworkopen.2022.51348 PMC9857138

[7] Kummerow KL, Du L, Penson DF, Shyr Y, Hooks MA (2015) Nationwide trends in mastectomy for early-stage breast cancer. JAMA Surg 150:9–16 25408966 10.1001/jamasurg.2014.2895

[8] Alaofi RK, Nassif MO, Al-Hajeili MR (2018) Prophylactic mastectomy for the prevention of breast cancer: review of the literature. Avicenna J Med 8:67–77 30090744 10.4103/ajm.AJM_21_18 PMC6057165

[9] Metcalfe KA, Birenbaum-Carmeli D, Lubinski J et al (2008) International variation in rates of uptake of preventive options in BRCA1 and BRCA2 mutation carriers. Int J Cancer 122:2017–2022 18196574 10.1002/ijc.23340 PMC2936778

[10] Lazow SP, Riba L, Alapati A, James TA (2019) Comparison of breast-conserving therapy vs mastectomy in women under age 40: national trends and potential survival implications. Breast J 25:578–584 31090168 10.1111/tbj.13293

[11] Dragun AE, Huang B, Tucker TC, Spanos WJ (2012) Increasing mastectomy rates among all age groups for early stage breast cancer: a 10-year study of surgical choice. Breast J 18:318–325 22607016 10.1111/j.1524-4741.2012.01245.x

[12] Garcia-Etienne CA, Tomatis M, Heil J et al (2013) Fluctuating mastectomy rates across time and geography. Ann Surg Oncol 20:2114–2116 23640480 10.1245/s10434-013-2982-x

[13] Shah JK, Amakiri UO, Cevallos P et al (2024) Updated trends and outcomes in autologous breast reconstruction in the United States, 2016–2019. Ann Plast Surg 92:e1–e13 38320006 10.1097/SAP.0000000000003764

[14] Masoomi H, Hanson SE, Clemens MW, Mericli AF (2021) Autologous breast reconstruction trends in the United States: using the Nationwide Inpatient Sample database. Ann Plast Surg 87:242–247 33443887 10.1097/SAP.0000000000002664

[15] Damen TH, de Bekker-Grob EW, Mureau MA et al (2011) Patients’ preferences for breast reconstruction: a discrete choice experiment. J Plast Reconstr Aesthet Surg 64:75–83 20570232 10.1016/j.bjps.2010.04.030

[16] Hall SE, Holman CD (2003) Inequalities in breast cancer reconstructive surgery according to social and locational status in Western Australia. Eur J Surg Oncol 29:519–525 12875859 10.1016/s0748-7983(03)00079-9

[17] Soon PS, Ruban S, Mo HTJ et al (2019) Understanding patient choices regarding breast reconstruction after mastectomy for breast cancer. Support Care Cancer 27:2135–2142 30251065 10.1007/s00520-018-4470-0

[18] Roy N, Downes MH, Ibelli T et al (2024) The psychological impacts of post-mastectomy breast reconstruction: a systematic review. Ann Breast Surg 8:19 10.21037/abs-23-33 PMC11296521 39100730

[19] Cordeiro PG (2008) Breast reconstruction after surgery for breast cancer. N Engl J Med 359:1590–1601 18843123 10.1056/NEJMct0802899

[20] Zehra S, Doyle F, Barry M, Walsh S, Kell MR (2020) Health-related quality of life following breast reconstruction compared to total mastectomy and breast-conserving surgery among breast cancer survivors: a systematic review and meta-analysis. Breast Cancer 27:534–566 32162181 10.1007/s12282-020-01076-1

[21] Shiraishi M, Sowa Y, Tsuge I, Kodama T, Inafuku N, Morimoto N (2022) Long-term patient satisfaction and quality of life following breast reconstruction using the BREAST-Q: a prospective cohort study. Front Oncol 12:815498 35692774 10.3389/fonc.2022.815498 PMC9178786

[22] Speck NE, Grufman V, Farhadi J (2023) Trends and innovations in autologous breast reconstruction. Arch Plast Surg 50:240–247 37256033 10.1055/s-0043-1767788 PMC10226796

[23] Hamdi M, Kapila AK, Waked K (2023) Current status of autologous breast reconstruction in Europe: how to reduce donor site morbidity. Gland Surg 12:1760–1773 38229849 10.21037/gs-23-288 PMC10788572

[24] Garza 3rd R, Ochoa O, Chrysopoulo M (2021) Post-mastectomy breast reconstruction with autologous tissue: current methods and techniques. Plast Reconstr Surg Glob Open 9:e3433 33680677 10.1097/GOX.0000000000003433 PMC7929567

[25] Joo JH, Ki Y, Kim W et al (2021) Pattern of local recurrence after mastectomy and reconstruction in breast cancer patients: a systematic review. Gland Surg 10:2037–2046 34268088 10.21037/gs-21-15 PMC8258883

[26] Buchanan CL, Dorn PL, Fey J et al (2006) Locoregional recurrence after mastectomy: incidence and outcomes. J Am Coll Surg 203:469–474 17000389 10.1016/j.jamcollsurg.2006.06.015

[27] Kaidar-Person O, Poortmans P, Offersen BV et al (2020) Spatial location of local recurrences after mastectomy: a systematic review. Breast Cancer Res Treat 183:263–273 32661665 10.1007/s10549-020-05774-4

[28] Rebbeck, Friebel T TR, Lynch HT et al (2004) Bilateral prophylactic mastectomy reduces breast cancer risk in BRCA1 and BRCA2 mutation carriers: the PROSE Study Group. J Clin Oncol 22:1055–1062 14981104 10.1200/JCO.2004.04.188

[29] Meijers-Heijboer H, van Geel B, van Putten WL et al (2001) Breast cancer after prophylactic bilateral mastectomy in women with a BRCA1 or BRCA2 mutation. N Engl J Med 345:159–164 11463009 10.1056/NEJM200107193450301

[30] Kaas R, Verhoef S, Wesseling J et al (2010) Prophylactic mastectomy in BRCA1 and BRCA2 mutation carriers: very low risk for subsequent breast cancer. Ann Surg 251:488–492 20134318 10.1097/SLA.0b013e3181c3c36d

[31] Al-Himdani S, Timbrell S, Tan KT, Morris J, Bundred NJ (2016) Prediction of margin involvement and local recurrence after skin-sparing and simple mastectomy. Eur J Surg Oncol 42:935–941 27256869 10.1016/j.ejso.2016.04.055

[32] Woitek R, Pfeiler G, Farr A et al (2018) MRI-based quantification of residual fibroglandular tissue of the breast after conservative mastectomies. Eur J Radiol 104:1–7 29857853 10.1016/j.ejrad.2018.04.028 PMC7547401

[33] Zippel D, Tsehmaister-Abitbol V, Rundstein A et al (2015) Magnetic resonance imaging (MRI) evaluation of residual breast tissue following mastectomy and reconstruction with silicone implants. Clin Imaging 39:408–411 25680501 10.1016/j.clinimag.2014.12.014

[34] Giannotti DG, Hanna SA, Cerri GG, Barbosa Bevilacqua JL (2018) Analysis of skin flap thickness and residual breast tissue after mastectomy. Int J Radiat Oncol Biol Phys 102:82–91 30102208 10.1016/j.ijrobp.2018.05.023

[35] Sessa C, Balmaña J, Bober SL et al (2023) Risk reduction and screening of cancer in hereditary breast-ovarian cancer syndromes: ESMO clinical practice guideline. Ann Oncol 34:33–47 36307055 10.1016/j.annonc.2022.10.004

[36] Loibl S, André F, Bachelot T et al (2024) Early breast cancer: ESMO clinical practice guideline for diagnosis, treatment and follow-up. Ann Oncol 35:159–182 38101773 10.1016/j.annonc.2023.11.016

[37] (NCCN®). NCCN (2024) NCCN clinical practice guidelines in oncology (NCCN guidelines®): breast cancer. Version 6.2024. Available via NCCN. https://www.nccn.org/guidelines/guidelines-detail?category=1&id=1419

[38] Kaidar-Person O, Boersma LJ, Poortmans P et al (2020) Residual glandular breast tissue after mastectomy: a systematic review. Ann Surg Oncol 27:2288–2296 32390098 10.1245/s10434-020-08516-4

[39] Hosmer DW, Lemeshow S (2004) Logistic regression. Applied. Wiley

[40] Kaplan EL, Meier P (1958) Nonparametric Estimation from Incomplete Observations. J Am Stat Assoc 53:457–481

[41] Mantel N (1966) Evaluation of survival data and two new rank order statistics arising in its consideration. Cancer Chemother Rep 50:163–170 5910392

[42] Cox DR (1972) Regression models and life-tables. J R Stat Soc B (Methodological) 34:187–220

[43] Kulturoglu MO, Aydin F, Sagdic MF et al (2025) The impact of changes in breast density over time on breast cancer risk. Sci Rep 15:23900 40615548 10.1038/s41598-025-09315-1 PMC12227740

[44] Chen JH, Chang YC, Chang D et al (2011) Reduction of breast density following tamoxifen treatment evaluated by 3-D MRI: preliminary study. Magn Reson Imaging 29:91–98 20832226 10.1016/j.mri.2010.07.009 PMC3005955

[45] Deutschmann C, Singer CF, Gschwantler-Kaulich D et al (2023) Residual fibroglandular breast tissue after mastectomy is associated with an increased risk of a local recurrence or a new primary breast cancer. BMC Cancer 23:281 36978031 10.1186/s12885-023-10764-y PMC10044359

[46] Papassotiropoulos B, Güth U, Chiesa F et al (2019) Prospective evaluation of residual breast tissue after skin- or nipple-sparing mastectomy: results of the SKINI-trial. Ann Surg Oncol 26:1254–1262 30830538 10.1245/s10434-019-07259-1

[47] Gui GP, Behranwala KA, Abdullah N et al (2004) The inframammary fold: contents, clinical significance and implications for immediate breast reconstruction. Br J Plast Surg 57:146–149 15037170 10.1016/j.bjps.2003.11.030

[48] Carlson GW, Grossl N, Lewis MM, Temple JR, Styblo TM (1996) Preservation of the inframammary fold: What are we leaving behind? Plast Reconstr Surg 98:447–450 8700979 10.1097/00006534-199609000-00012

[49] Kroll SS, Ames F, Singletary SE, Schusterman MA (1991) The oncologic risks of skin preservation at mastectomy when combined with immediate reconstruction of the breast. Surg Gynecol Obstet 172:17–20 1985335

[50] Kaidar-Person O, Sklair-Levy M, Anaby D et al (2024) Residual breast tissue after mastectomy and reconstruction: a substudy of the spatial location of breast cancer local recurrence after mastectomy (SECRET) project. Eur J Surg Oncol 50:108607 39191132 10.1016/j.ejso.2024.108607

[51] Christine D, Christian SF, Ricarda K et al (2024) Risk factors for residual fibroglandular breast tissue following a mastectomy - an overview and retrospective cohort study. BMC Cancer 24:856 39026150 10.1186/s12885-024-12491-4 PMC11256640

[52] Courtney D, Davey MG, Moloney BM et al (2022) Breast cancer recurrence: factors impacting occurrence and survival. Irish J Med Sci 191:2501–2510 35076871 10.1007/s11845-022-02926-x PMC9671998

[53] Grinstein O, Krug B, Hellmic M et al (2019) Residual glandular tissue (RGT) in BRCA1/2 germline mutation carriers with unilateral and bilateral prophylactic mastectomies. Surg Oncol 29:126–133 31196476 10.1016/j.suronc.2019.04.009

[54] Wang J, Tang Y, Jing H et al (2020) Risk stratification for prediction of locoregional recurrence in patients with pathologic T1-2N0 breast cancer after mastectomy. BMC Cancer 20:1132 33228588 10.1186/s12885-020-07594-7 PMC7685539

[55] Handel N, Silverstein MJ, Waisman E, Waisman JR (1990) Reasons why mastectomy patients do not have breast reconstruction. Plast Reconstr Surg 86:1118–1122 2243854

[56] Mohrmann S, Kolberg L, Jäger B et al (2023) Impact of surgical variables on residual glandular tissue in risk-reducing mastectomies: results of a retrospective monocentric study from a Centre of the German Consortium for hereditary breast and ovarian cancer. Eur J Surg Oncol 49:107031 37683424 10.1016/j.ejso.2023.107031

[57] Dietzel F, Kolberg L, Vesper AS et al (2023) Factors influencing residual glandular breast tissue after risk-reducing mastectomy in genetically predisposed individuals detected by MRI mammography. Cancers (Basel) 15:829 36765786 10.3390/cancers15030829 PMC9913581

[58] Torresan RZ, dos Santos CC, Okamura H, Alvarenga M (2005) Evaluation of residual glandular tissue after skin-sparing mastectomies. Ann Surg Oncol 12:1037–1044 16244800 10.1245/ASO.2005.11.027

[59] Fritsch H, Tumeltshammer R, Hachenberg J et al (2024) Comparison of the preoperative MRI evaluation of glandular tissue in subcutaneous mastectomy and its influence on the implant volume. Cancer Diagn Progn 4:599–604 39238620 10.21873/cdp.10369 PMC11372688

[60] Kaidar-Person O, Faermann R, Polikar D et al (2024) A BRILLIANT-BRCA study: residual breast tissue after mastectomy and reconstruction. Breast Cancer Res Treat 208:359–367 38980506 10.1007/s10549-024-07425-4 PMC11455724

[61] Dietzel M, Wenkel E, Hammon M et al (2019) Does higher field strength translate into better diagnostic accuracy? A prospective comparison of breast MRI at 3 and 1.5 Tesla. Eur J Radiol 114:51–56 31005176 10.1016/j.ejrad.2019.02.033

[62] Rothman KJ (1986) Modern epidemiology. Little, Brown and Company, Boston/Toronto

