# Supplementary material for: Detection of residual fibroglandular tissue on breast MRI and associated factors in women treated with mastectomy and DIEP flap breast reconstruction
Source: Insights Imaging. 2026 Aug 26;17:208. doi: 10.1186/s13244-026-02382-6 (PMC13518751; doi:10.1186/s13244-026-02382-6)
Supplement: Supplementary file 1 [file 13244_2026_2382_MOESM1_ESM.docx]

**Appendix 1**. Overview of MR protocols for standard breast MRI between 2007 – 2022

MRI 1: Philips Intera 1.5T, protocols for standard breast MRI between 2007 and 2016 (2014 and 2015 not applicable).

|  | **2007** | **2008** | **2009** | **2010** |
| --- | --- | --- | --- | --- |
| **Coil** | Sense body coil | Sense body coil | Sense body coil | Sense body coil |
| **Matrix** | 512 x 512 | 512 x 512 | 512 x 512 | 576 x 576 |
| **Pixel spacing (mm)** | 0.664^2^ | 0.820^2^ | 0.742^2^ | 0.605^2^ |
| **Field of View (mm)** | 340^2^ | 420^2^ | 380^2^ | 349^2^ |
| **In-plane resolution (mm)** | 3.0^2^ | 3.0^2^ | 3.0^2^ | 3.0^2^ |
| **Repetition time (msec)** | 7008 | 4079 | 5626 | 3004 |
| **Echo time (msec)** | 120 | 120 | 110 | 110 |
| **Flip angle (degrees)** | 90 | 90 | 90 | 90 |
| **Echo train length** | 30 | 35 | 33 | 29 |
| **Slice thickness (mm)** | 3.0 | 3.0 | 3.0 | 3.0 |
|  | **2011** | **2012** | **2013** | **2016** |
| **Coil** | Sense breast 16-channel | Sense breast 16-channel | Sense breast 16-channel | Sense breast 16-channel |
| **Matrix** | 384 x 384 | 384 x 384 | 432 x 432 | 432x432 |
| **Pixel spacing (mm)** | 0.959^2^ | 0.959^2^ | 0.787^2^ | 0,787^2^ |
| **Field of View (mm)** | 368^2^ | 368^2^ | 340^2^ | 340^2^ |
| **In-plane resolution (mm)** | 1.0^2^ | 1.0^2^ | 1.0^2^ | 1.0^2^ |
| **Repetition time (msec)** | 2000 | 2000 | 2000 | 2000 |
| **Echo time (msec)** | 205 | 205 | 258 | 258 |
| **Flip angle (degrees)** | 90 | 90 | 90 | 90 |
| **Echo train length** | 80 | 80 | 96 | 96 |
| **Slice thickness (mm)** | 2.0 | 2.0 | 2.0 | 2.0 |

MRI 2: Philips Ingenia 1.5T, protocols for standard breast MRI between 2014 and 2022 (2007 to 2013 not applicable).

|  | **2014** | **2015** | **2016** | **2017** | **2018** |
| --- | --- | --- | --- | --- | --- |
| **Coil** | Sense breast 16-channel | Sense breast 16-channel | Sense breast 16-channel | Sense breast 16-channel | Sense breast 16-channel |
| **Matrix** | 432 x 432 | 400x400 | 400x400 | 432x432 | 432x432 |
| **Pixel spacing (mm)** | 0.843^2^ | 0.854^2^ | 0,854^2^ | 0,787^2^ | 0,787^2^ |
| **Field of View (mm)** | 364^2^ | 341^2^ | 341^2^ | 340^2^ | 340^2^ |
| **In-plane resolution (mm)** | 1.0^2^ | 1.0^2^ | 1.0^2^ | 1.0^2^ | 1.0^2^ |
| **Repetition time (msec)** | 2000 | 2000 | 2000 | 2000 | 2000 |
| **Echo time (msec)** | 218 | 221 | 221 | 222 | 222 |
| **Flip angle (°)** | 90 | 90 | 90 | 90 | 90 |
| **Echo train length** | 95 | 95 | 95 | 95 | 95 |
| **Slice thickness (mm)** | 2.0 | 2.0 | 2.0 | 2.0 | 2.0 |
|  | **2019** | **2020** | **2021** | **2022** |  |
| **Coil** | Sense breast 16-channel | Sense breast 16-channel | Sense breast 16-channel | Sense breast 16-channel |  |
| **Matrix** | 528x528 | 432x432 | 432x432 | 432x432 |  |
| **Pixel spacing (mm)** | 0,707^2^ | 0,787^2^ | 0,787^2^ | 0,787^2^ |  |
| **Field of View (mm)** | 373^2^ | 340^2^ | 340^2^ | 340^2^ |  |
| **In-plane resolution (mm)** | 1.0^2^ | 1.0^2^ | 1.0^2^ | 1.0^2^ |  |
| **Repetition time (msec)** | 2000 | 2000 | 2000 | 2000 |  |
| **Echo time (msec)** | 222 | 222 | 222 | 222 |  |
| **Flip angle (°)** | 90 | 90 | 90 | 90 |  |
| **Echo train length** | 95 | 95 | 95 | 95 |  |
| **Slice thickness (mm)** | 2.0 | 2.0 | 2.0 | 2.0 |  |

MRI 3: Philips Achieva 3.0T, protocols for standard breast MRI in 2012 and 2013 (2007 to 2011 and 2014 to 2022 not applicable).

|  | **2012** | **2013** |
| --- | --- | --- |
| **Coil** | Sense breast 16-channel | Sense breast 16-channel |
| **Matrix** | 576 x 576 | 640 x 640 |
| **Pixel spacing (mm)** | 0.590^2^ | 0.547^2^ |
| **Field of View (mm)** | 340^2^ | 350^2^ |
| **In-plane resolution (mm)** | 2.0^2^ | 2.0^2^ |
| **Repetition time (msec)** | 5294 | 5294 |
| **Echo time (msec)** | 100 | 100 |
| **Flip angle (°)** | 90 | 90 |
| **Echo train length** | 27 | 27 |
| **Slice thickness (mm)** | 2.0 | 2.0 |
