# Supplementary material for: Detection of residual fibroglandular tissue on breast MRI and associated factors in women treated with mastectomy and DIEP flap breast reconstruction
Source: Insights Imaging. 2026 Aug 26;17:208. doi: 10.1186/s13244-026-02382-6 (PMC13518751; doi:10.1186/s13244-026-02382-6)
Supplement: Supplementary file 2 [file 13244_2026_2382_MOESM2_ESM.docx]

**Appendix 2.** Score form residual fibroglandular tissue on Breast MRI

Date assessment:……………………………

Assessor: ………………………………………

| **Date MRI** |  |
| --- | --- |
| **Residual fibroglandular tissue 1** | Diameter (mm): |
|  | Confidence scale (1-5): |
| **Residual fibroglandular tissue 2** | Diameter (mm): |
|  | Confidence scale (1-5): |
| **Residual fibroglandular tissue 3** | Diameter (mm): |
|  | Confidence scale (1-5): |
| **Residual fibroglandular tissue 4** | Diameter (mm): |
|  | Confidence scale (1-5): |
| **Residual fibroglandular tissue 5** | Diameter (mm): |
|  | Confidence scale (1-5): |
| **Residual fibroglandular tissue 6** | Diameter (mm): |
|  | Confidence scale (1-5): |
| **Residual fibroglandular tissue 7** | Diameter (mm): |
|  | Confidence scale (1-5): |
| **Residual fibroglandular tissue 8** | Diameter (mm): |
|  | Confidence scale (1-5): |
| **Residual fibroglandular tissue 9** | Diameter (mm): |
|  | Confidence scale (1-5): |
| **Residual fibroglandular tissue 10** | Diameter (mm): |
|  | Confidence scale (1-5): |

Confidence scale: 1 = definitely no breast tissue, 5 = definitely breast tissue

Right:	Left:
